# IgM-mediated epidermolysis bullosa acquisita

**DOI:** 10.1016/j.jdcr.2024.01.004

**Published:** 2024-01-24

**Authors:** Sidney Harrison, Vita Mayes, Conrad Brimhall, Roy King

**Affiliations:** a DeBusk College of Osteopathic Medicine, Lincoln Memorial University, Knoxville, Tennessee; b Lakeway Dermatology Associates, Morristown, Tennessee; c Dermatopathology Partners, Knoxville, Tennessee; d Department of Pathology, University of Tennessee, Knoxville, Tennessee

**Keywords:** bullous pemphigoid, case report, dermatitis herpetiformis, EBA, epidermolysis bullosa acquisita, porphyria cutanea tarda

## Introduction

Epidermolysis bullosa acquisita (EBA) is a rarely reported autoimmune subepidermal bullous disease that is typically identified as skin fragility with the formation of tense vesicles and bullae that rupture leaving erosions. EBA most commonly affects areas associated with trauma such as hands, feet, elbows, and back. The first cases of EBA were described in 1895 as 2 adults with acquired skin fragility by Elliot.^1^ EBA is characterized by the deposition of IgG directed against the anchoring fibrils in the sublamina densa of the basement zone. IgM, IgA, and complement may occasionally be found at the basement zone. We report a rare case of a patient with mechanobullous type EBA with only IgM class antibody identified along the basement membrane zone. To our knowledge, there are only 2 previously reported cases of IgM-mediated EBA.

## Case report

**Fig 1:**
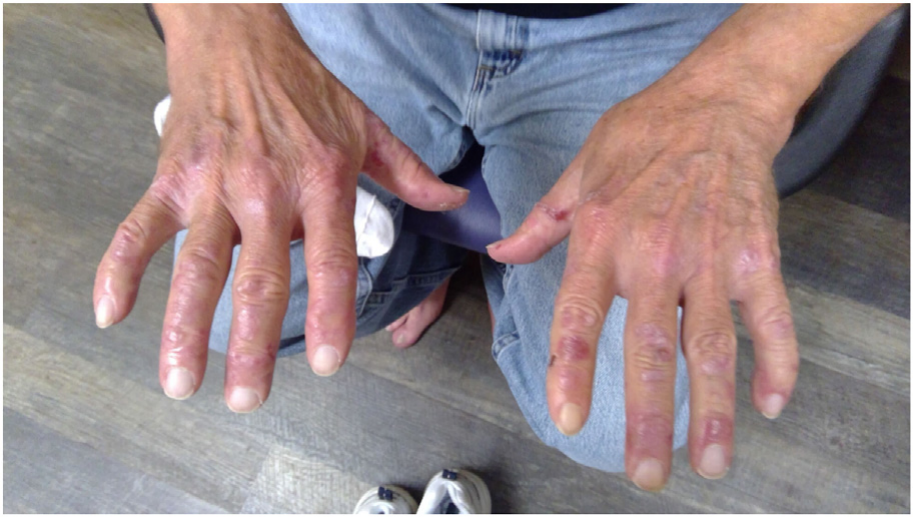
Demonstration of blisters with various stages of healing and scarring on bilateral aspect of the hands.

**Fig 2:**
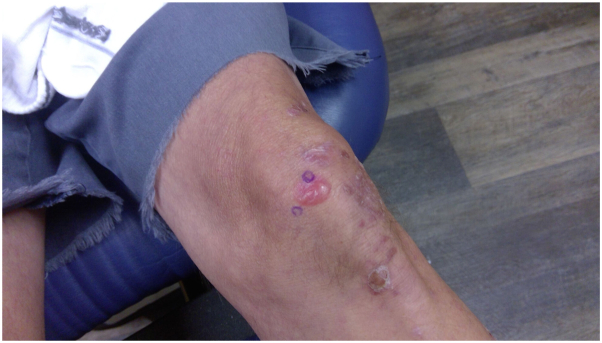
Demonstration of blisters with various stages of healing and scarring on left knee.

A 67-year-old man Caucasian patient presented with a 1-year history of onychoschizia involving all fingernails. This was accompanied by the development of severe blisters involving the patient’s hands and fingers, elbows, knees, feet, and back of the scalp. The skin would tear easily, and blisters would form in areas of friction. Physical examination revealed multiple bullae and vesicles in various stages of formation and healing located predominately on the elbows, dorsal and palmar aspect of the hands and fingers, knees, shins, toes, dorsal aspect of the feet, and back of the scalp (Figs 1 and 2). Scarring and milia formation was noted at the sites of old lesions. His relevant past history included chronic anemia of unknown cause treated with iron supplements, 1 pack/day smoking history and 8 beer/day drinking habit. He had actinic keratoses treated with liquid nitrogen and has a history of basal cell and squamous cell carcinomas.

**Fig 3:**
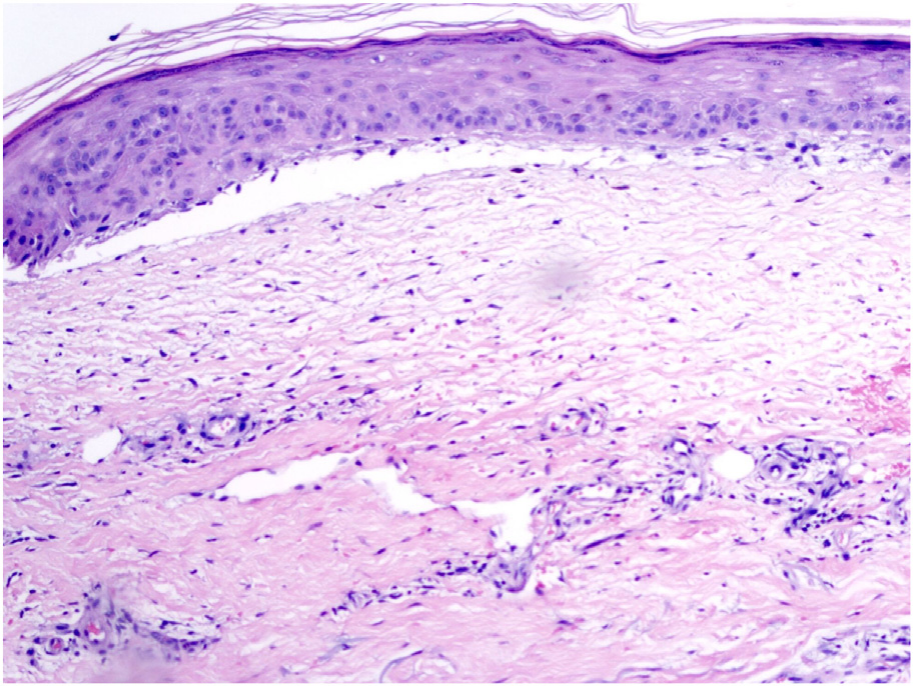
Cell poor unilocular subepidermal blister (Hematoxylin-eosin stain; original magnification: ×20).

**Fig 4:**
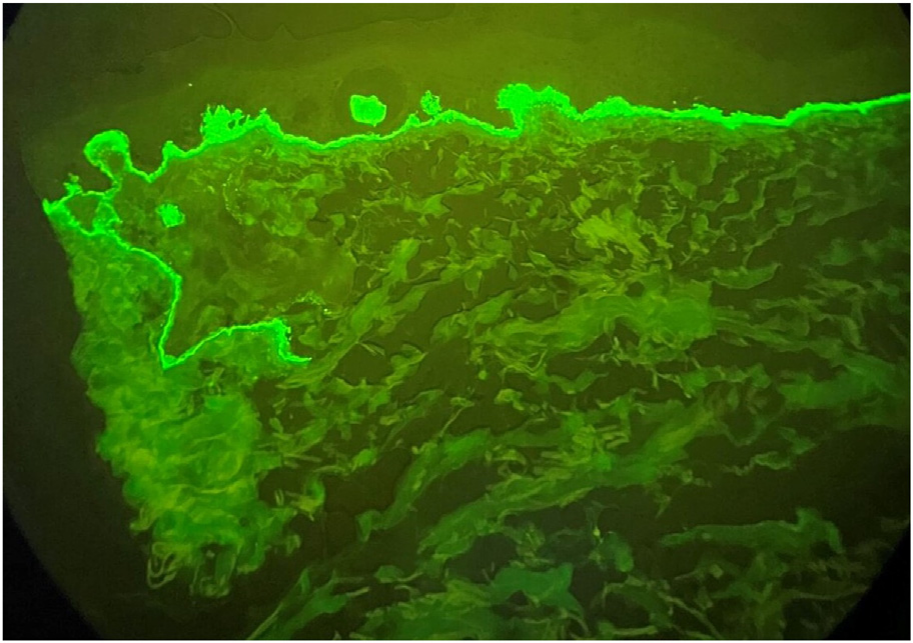
Linear IgM basement membrane staining (4+) on direct immunofluorescence staining (Original magnification: ×40).

**Fig 5:**
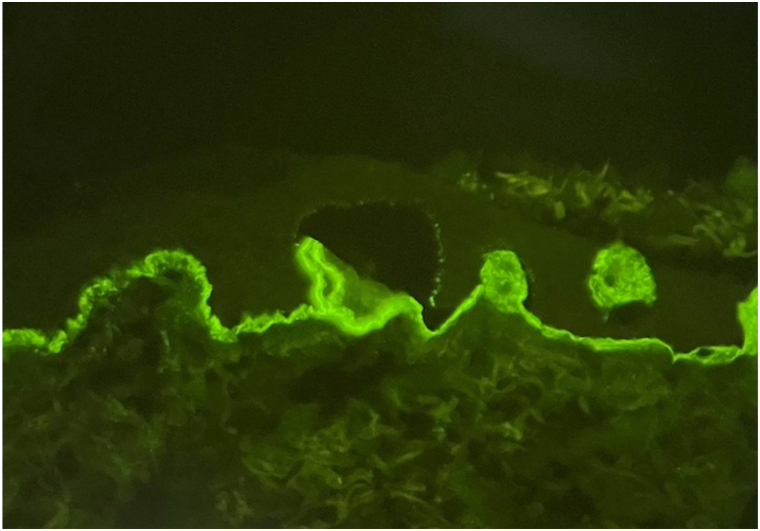
Salt split immunofluorescence study demonstrating a partial subepidermal blister with 4 IgM staining to the floor of the blister.

Punch biopsy revealed a pauci-inflammatory subepidermal vesicle. Only scattered perivascular lymphocytes were present. Eosinophils and papillary dermal microabscess formation were not present (Fig 3). Direct immunofluorescence (DIF) studies demonstrated 4+ linear IgM (Fig 4) basement membrane staining. No deposits of IgG, IgA, and complement were detected along the basement membrane or in the epidermis. Given these findings, the direct immunofluorescence specimen was melted down and the specimen was incubated in 5 mL of NaCl (1mol/L) at 4 °C for 24 hours. The epidermis was teased from the dermis with forceps and direct immunofluorescence studies with IgM demonstrated a partial subepidermal blister with 4+ IgM staining on the floor of the blister (Fig 5). Twenty-four hour urine collection revealed porphyrin levels within normal limits. ELISA Antibody testing indicated no autoantibodies for collagen type VII or BP180 and BP230.

The blistering only marginally improved with dapsone 100 mg every day and the patient continued to get blister formation with mechanical friction, particularly on hands and fingers. Everyday activities such as opening jars and removing adhesive bandages would cause skin tears and blister formation. Prednisone 20 mg every day was added to the treatment regimen, however, the patient only tolerated this addition for 1 week and was discontinued. Mycophenolate mofetil 1000 mg twice a day was added to the dapsone with minimal improvement. Then a short course of colchicine 0.6 daily was added with no effect. All treatments were discontinued and a trial of Dupixent 600 mg subcutaneous loading dose was administered with subsequent 300 mg subcutaneous maintenance doses every 2 weeks. However, the patient was noncompliant after a month and a half and discontinued treatment with no noticeable improvement.

## Discussion

EBA is a chronic autoimmune subepidermal blistering disease caused by circulating autoantibodies against NC1 domain of type VII collagen. Type VII collagen is an adhesion molecule in the epidermal basement membrane and the primary constituent of the anchoring fibrils providing attachment of the epidermis to the dermis. EBA is most associated with type VII collagen autoantibodies, specifically the IgG subtype and is seen as linear basement deposits at the dermoepidermal junction.^2^ In addition, with indirect immunofluorescence on salt split skin tissue, antibody deposits will be seen on the dermal side of the blister. EBA has 2 main subtypes: inflammatory and mechanobullous (classic, noninflammatory).^3^ As seen in our patient, the mechanobullous type is characterized by increased skin fragility with blisters or erosions at sites of trauma and nail dystrophy is often a comorbid condition. The IgA EBA subtype is also observed but is seen more often in the nonmechanobullous EBA and when mucosal surfaces are involved.^4^ The IgM EBA subtype, as seen in our case is rare with only 2 other previous cases reported. Although the clinical, histopathology, and DIF findings of the other 3 reported cases of IgM-EBV are similar, the phenotype described in our patient was more severe in nature and more treatment resistant.^5^^,^^6^

EBA must be differentiated from other disorders as there may be clinical overlap with other blistering diseases such as porphyria cutanea tarda, bullous pemphigoid, and dermatitis herpetiformis. In our case, the normal 24-hour urine porphyrin levels ruled out porphyria cutanea tarda. Dermatitis herpetiformis was excluded as the symmetric pruritic papulovesicular eruption on extensor surfaces was absent as was the histologic picture of neutrophilic microabscess formation and DIF IgA basement membrane and papillary dermal staining. The paucicellular subepidermal blister, lack of IgG and C3 DIF basement staining together with negative autoantibody testing for BP180 and BP230 would rule out bullous pemphigoid in our patient.

Our case highlights the importance of DIF in detecting immunobullous diseases as well as differentiating EBA from other autoimmune subepidermal bullous diseases. Standard ELISA serum testing for antibody to type VII collagen and regular indirect immunofluorescence would be negative as both tests use labeled antihuman IgG and do not specifically test for IgM in serum. Our case is similar to the 2 previous cases of IgM-mediated EBA and may represent a subtype of that disease with mechanobullous features. A significant difference was our patient’s refractory response to treatment and further study will be needed to provide data-driven treatment for this rare subset.

## Conflicts of interest

None disclosed.
